# The role of family medicine and primary health care and its impact on the climate crisis

**DOI:** 10.4102/safp.v65i1.5658

**Published:** 2023-02-10

**Authors:** Indiran Govender

**Affiliations:** 1 Department Family Medicine and Primary Health Care, Faculty of Health Sciences, Sefako Makgatho Health Sciences University, Pretoria, South Africa

The 2022 South African Academy of Family Practice (SAAFP) conference plenary on the climate change highlighted the global warming crisis. The role of family medicine and primary health care in respect of the climate crisis is crucial now more than ever. The environmental crisis was further highlighted at the COP27 (Conference of the Parties) meeting in November 2022 in Egypt. This environmental crisis is the greatest threat to all life on earth.^1^

Primary health care (PHC) workers including Family Medicine professionals are the frontline workers of health care. The climate change crisis is becoming a health threat and thus cannot be ignored. Primary health care roles involving comprehensive PHC, community engagement and empowerment should be emphasised in regard to the effects of global warming. Primary health care is being re-engineered by the implementation of the National Health Insurance (NHI), but unfortunately, there is minimal focus on addressing climate changes and health. Focus should be on the link between community health challenges such as increased lung disease and gastrointestinal conditions leading to dehydration and increased risk of malnutrition in a community and excessive greenhouse gas emissions leading to more air pollution, droughts and climate-related disasters such as fires, floods and heat waves, which will impact community members’ lives and food production.^1,2,3^ Global warming can result in large scale societal disruption, worsen poverty by increasing inequities and result in people migrating because of scarcity of resources, resulting in a decrease in continuity of care and poor health.^1,2,4^ Primary health care services, facilities, human resources and supply chains could be disrupted by growing environmental changes, preventing the possibility of providing comprehensive, coordinated services.^5,6^ The recent heat waves in Europe, floods in KwaZulu-Natal and Pakistan, and Hurricane Ian in Florida (United States) are examples of things to come.^1,3,4,5^

Many aspects of adapting to climate change and reducing its impact can only be addressed by policymakers.^7,8,9^ Policymakers thus need to understand the impact of climate change on housing, food prices, exposure to severe weather events, vector- and water-borne diseases. The relationship between climate change and the changing disease profiles such as the increase of chronic obstructive pulmonary disease, dermatological cancers, and other chronic diseases of lifestyle should be considered.^6,10,11^ Policymakers should also consider that the health response to global warming will be delivered by PHC workers, who are also affected by climate-related threats and that PHC providers will have to contribute to both adapting to climate change and reducing the impact by providing health promotion measures and integrated health services.^7,11^

While policymakers have the major role to play, we can still contribute to making our community and policymakers aware of the threat to health that climate change bring. In the United Kingdom, nurses are addressing climate change by increasing awareness and advocating for and implementing climate adaptation measures.^7,8^

Multisectoral acknowledgement and action are needed in this urgent matter, but unfortunately it seems that when this situation is acknowledged at all, it appears that few decision-makers are actually doing anything to address this.^3,12^ At a meeting of the Group of 20 industrialised countries, only two referred to climate change: France recognised that global warming will contribute to an increase in vector-borne diseases while Indonesia acknowledged the need for strategies to adapt to health effects of global warming.^4,8^ Other countries do not discuss the impact of the climate crisis on primary care services or essential health functions or the potential contribution of community engagement to global warming. This context illustrates the urgent need that other countries also recognise climate change as a potential health challenge, especially when it comes to PHC. After this recognition, the next step would be to plan to address the situation by adapting PHC services to influence the community’s health and not just predict the health effects on the community because of climate change.^13^

Climate adaptation plans must address the primary health care component with multisectoral action, particularly with water, sanitation, hygiene, nutrition and agricultural interventions.^4,8,13^ South Africa called for increasing existing public health interventions but failed to mention specific plans. However, India and the United States included specific plans for community engagement by doing community research and focusing on building resilience.^7,13^

The World Health Organization’s vision for PHCs includes a responsive, efficient and cost-effective service delivery as well as equitable outcomes. Changing disease patterns and the other effects of climate change affect mostly low-income countries and vulnerable populations located in the southern hemisphere, many of which contribute least to carbon emissions.^3,13^ High-income countries have a responsibility to do more to reduce their share of carbon pollution.

Primary health care must be purposefully adapted to counter these problems of climate change. Primary health care adaptation and improvement offer opportunities to improve population health and well-being, and to empower populations to implement positive changes to both health and the climate crisis.^13^ Engaging with the community and providing public health, preventive and curative health services require community empowerment and mobilisation. Thus, primary health care represents an opportunity to build on the synergies, strengthen and mobilise communities to deliver the necessary asset requirements for health today and for future generations.^7,8^ The PHC level is where health and community action meet most closely, which includes going into communities for outreach programmes and health promotion and awareness campaigns.^7^ This community–health interface provides an acceptable access to social capital and resilience to the climate crisis.^7,13^ Many social participation programmes for health currently exist, and these provide the potential to foster community engagement on the climate crisis more holistically.^7,8^

Policymakers from high-income, high-carbon emitting countries could have a large impact on climate change by working towards becoming carbon zero by 2040 as intended by the National Health Service. Recommendations for policymakers at the national level include: revise existing health plans to include a robust situation analysis of climate change and health implications, designing PHC-based approaches to address the gaps; ensure the heath sector is actively engaged in climate crisis decision-making; include PHC-based approaches across the life course and analysis of interventions for the climate crisis in national plans, commitments and budget allocations.^5,10,11^ Locally produced plant-based diets and the use of renewable energy instead of fossil fuel-based energy should be considered.^4,7^ At the regional levels: provide normative guidance on PHC that directly addresses threats to populations, communities and systems posed by the climate crisis; continue to build a focus on PHC and climate-related issues through the global action plan for healthier lives.^5,10,11^ Focus should be on strengthening governance structures across sectors to help PHC health systems meet the impact of climate change.^13^

To reach the health-related sustainable development goal, greater convergence between leadership and community action on PHC and the climate crisis is required. There needs to be intersectoral collaboration with communities to provide unified and effective strategies to promote health and manage climate change.^13^ Thus, I feel that PHC and the climate crisis cannot be dealt with separately and the time for action is now!
